# Health data research on sudden cardiac arrest: perspectives of survivors and their next-of-kin

**DOI:** 10.1186/s12910-021-00576-9

**Published:** 2021-01-28

**Authors:** Marieke A. R. Bak, Rens Veeken, Marieke T. Blom, Hanno L. Tan, Dick L. Willems

**Affiliations:** 1 Section of Medical Ethics, Department of General Practice, Amsterdam, UMC, University of Amsterdam, Meibergdreef 9, 1105 AZ Amsterdam, The Netherlands; 2 Faculty of Medicine, Amsterdam, UMC, University of Amsterdam, Amsterdam, The Netherlands; 3 Department of Cardiology, Heart Center, Amsterdam, UMC, University of Amsterdam, Amsterdam, The Netherlands; 4 Netherlands Heart Institute, Utrecht, The Netherlands

**Keywords:** Biobanking, Health registries, Big data, Informed consent, Emergency medicine, Death, Research ethics, ESCAPE-NET

## Abstract

**Background:**

Consent for data research in acute and critical care is complex as patients become at least temporarily incapacitated or die. Existing guidelines and regulations in the European Union are of limited help and there is a lack of literature about the use of data from this vulnerable group. To aid the creation of a patient-centred framework for responsible data research in the acute setting, we explored views of patients and next-of-kin about the collection, storage, sharing and use of genetic and health-related data for observational research.

**Methods:**

We conducted qualitative interviews (n = 19) with Dutch sudden cardiac arrest survivors who donated clinical and socio-economic data and genetic samples to research. We also interviewed their next-of-kin. Topics were informed by ethics literature and we used scenario-sketches to aid discussion of complex issues.

**Results:**

Sudden cardiac arrest survivors displayed limited awareness of their involvement in health data research and of the content of their given consent. We found that preferences regarding disclosure of clinically actionable genetic findings could change over time. When data collection and use were limited to the medical realm, patients trusted researchers to handle data responsibly without concern for privacy or other risks. There was no consensus as to whether deferred consent should be explicitly asked from survivors. If consent is asked, this would ideally be done a few months after the event when cognitive capacities have been regained. Views were divided about the need to obtain proxy consent for research with deceased patients’ data. However, there was general support for the disclosure of potentially relevant post-mortem genetic findings to relatives.

**Conclusions:**

Sudden cardiac arrest patients’ donation of data for research was grounded in trust in medicine overall, blurring the boundary between research and care. Our findings also highlight questions about the acceptability of a one-time consent and about responsibilities of patients, researchers and ethics committees. Finally, further normative investigation is needed regarding the (continued) use of participants’ data after death, which is of particular importance in this setting. Our findings are thought to be of relevance for other acute and life-threatening illnesses as well.

## Background

The use of patient data for research carries great potential to improve care and decrease the burden of illness on patients and health care systems, especially given the advances in big data and machine learning and the rise of biobanking [1–4]. At the same time, the analysis of health data creates ethical and legal challenges particularly with regard to subjects’ informational privacy [5]. The ‘bigger’ (e.g., in terms of size, detail, variety, accessibility, or extent of potential uses) these data are, the more difficult it becomes to protect patients’ autonomy through measures like anonymization, if possible at all, and informed consent which presupposes understanding of the research [6]. Moreover, health data research may bring about group-level ethical issues relating to stigma and unfair discrimination [7, 8]. In this context, genomic data are often seen as particularly sensitive, since they are almost by definition personally identifiable and may reveal information about blood relatives [9–12].

In 2018, the European Union’s General Data Protection Regulation (GDPR) came into force to harmonise data protection rules across the EU and to provide data subjects with more control over their personal data than the earlier 1995 Directive [13]. The GDPR defines genetic, biometric and other health-related data as a special category of data (together with “personal data revealing racial or ethnic origin, political opinions, religious or philosophical beliefs, or trade union membership, […] or data concerning a natural person's sex life or sexual orientation” [Article 9]). Processing of these types of sensitive data is in principle prohibited, but one may appeal to exceptions such as informed consent or public interest. In the setting of acute and critical care there is a tension between data protection legislation and research since “it is not possible to ask for the patients’ informed consent to be enrolled in observational research at the point of admission to the hospital” (p. 59) [14]. Yet the use of large-scale patient data is necessary for acute conditions like sudden cardiac arrest (SCA) that have a diversity of causes and are often the first expression of underlying disease [15]. Disease-specific biobanks and registries are needed to obtain sufficiently large numbers of samples, and because the clinical data needed for this research (e.g., ambulance ECGs) are usually not collected routinely.

While there has been ample discussion of the issues around *interventional* research in people with acute and critical conditions, there is a dearth of literature about the responsible use of data from this patient group for *observational* studies [16]. Research using data from people with acute life-threatening conditions (e.g., stroke, SCA, traumatic brain injury, or acute respiratory failure due to infectious disease) brings about a number of ethical questions. For instance, is a consent waiver permissible to enable the use of data from patients who cannot give consent? While explicit informed consent has been the gold standard of clinical trial ethics, for registry and biobanking studies some jurisdictions allow ethics committees to waive the consent requirement or to enable opt-out mechanisms, under specific conditions like a requirement for anonymization [17]. In case of truly anonymous data, there is generally no obligation to obtain consent because these data do not fall under the scope of the GDPR. However, in emergency medicine, working with fully anonymized data is especially difficult due to the different data sources in the “chain of care” and the need to link these using personal identifiers. Anonymization is also problematic from another perspective: complete de-identification would make it impossible to return clinically relevant and actionable (genetic) research findings to subjects.

In addition, the acute and critical care setting is special because relatively many patients will not survive their sudden medical event or hospitalization. For SCA, the survival rate ranges from 3% to 23% across Europe [18]. However, there is no international consensus regarding the acceptability of post-mortem observational research without consent from next-of-kin, or about the conditions that should apply. The growing contribution of deceased persons to health research databases also brings to the fore questions about disclosure of individual genetic findings by researchers to relatives. Most hereditary arrhythmia syndromes and cardiomyopathies that increase the risk of SCA are inherited in an autosomal-dominant fashion (providing a 50% chance to be passed on to either sex). Disclosure of such findings can help to tailor preventive interventions but may also cause concerns among recipients about their health, the right not to know, and about privacy and decision-making within families [19, 20]. Should these individual research findings be reported posthumously to next-of-kin? And if a person has stated that family should not be informed of any clinically actionable genetic findings, should this wish be respected after his or her death? Current ethical guidelines and regulations, including the GDPR, do not provide the needed guidance on the post-mortem use of data for research [21, 22].

We think that, especially in the acute setting in which the decision-making capacity of critically ill participants is absent or at least very limited, it is important to obtain patient views on topics as informed consent and other relevant issues [23]. Therefore, in this paper we present our findings based on interviews with Dutch SCA survivors, and their next-of-kin, about the donation of their data to research, with the aim of contributing to an empirically informed, patient-centred ethical framework on data use in emergency care research, as well as to the broader health data privacy debate. Our study was situated within an SCA research group in the Netherlands where, apart from the GDPR, national data protection and civil law regulate the use of patient data for research. However, the ethical issues discussed are also of relevance to research in other countries and other acute settings.

## Methods

### Study design and setting

We performed a qualitative, semi-structured interview study in the Netherlands with patients who survived SCA and with their next-of-kin.^1^ The study was funded by a EU Horizon 2020 grant which had no role in the design or execution of the research. Our methodological orientation was one of empirical ethics [24] and we based our interview topics on previous literature research [16]. Patients who were interviewed had donated their data to the ARREST (AmsteRdam REesuscitation STudies) research project. This project, part of the European ESCAPE-NET consortium, is an ongoing registry that investigates causes and treatment of out-of-hospital SCA [25, 26]. Data for ARREST are collected through various data sources in the ‘chain of care’ such as ambulance services and hospitals. These sources provide information about pre-hospital treatment (resuscitation by citizen rescuers or ambulance personnel), in-hospital treatment and diagnosis, patients’ medical history and medication use, name and date of birth, (pseudo-anonymised) socio-economic data like household income, and biosamples including DNA from residual materials that were obtained for the sake of routine clinical care (blood, intubation tubes). Some data are collected without prospective consent, because they need to be saved quickly (e.g., ambulance ECGs will otherwise be overwritten). For other data, surviving patients are approached to ask for opt-in consent, three months or more after the resuscitation. Contact is initiated with a letter from the ambulance service after which, unless they opt out from being contacted, patients are approached with consent documentation by ARREST researchers. Until deferred consent has been obtained, the data are not used for the ARREST study. For genetic data, a separate consent option is provided. In the year 2016, a total of 1126 patients were eligible for ARREST (one-fifth alive at discharge). Of the survivors, 67% consented while 9% declined to give consent and the remainder were not reachable.

### Recruitment

Patients were eligible for an interview if they: were ≥18 years old at the time of interview; had previously consented to participate in ARREST; had declared that they could be approached again; were sufficiently fluent in the Dutch language to participate in the interview; and had a Cerebral Performance Category (CPC) score of 1 at hospital discharge after resuscitation, to increase the likelihood that they would be cognitively fit for an interview and for their consent to be informed. Eligible ARREST participants were purposively selected (as much as possible within the constraints of the sample) based on diversity in age, sex, ethnicity, and preferences regarding disclosure of individual genetic findings. They were approached by telephone to gauge their interest. Next-of-kin (spouses and blood relatives of ≥18 years old) were found through participating patients.

Information about the study and the informed consent forms were mailed to participants before the interview. Participants were given the option to receive and correct interview transcripts and, eventually, study results. The local Research Ethics Committee (REC) stated that approval for the interview study was not required under Dutch law.

### Interview guide and procedure

Based on practical experience and previous literature research [16], we developed an interview topic guide which was used for this study and to evaluate patients’ experiences with consent for the ARREST project (Additional file 1). This guide was pilot-tested on an experienced researcher and cardiologist in a role-play, discussed with the ARREST team, and shared with co-authors multiple times during the study. We re-evaluated the questions after each interview and changed their wordings or order, if needed, but left the main topics unchanged. An adapted, shorter version was used to interview next-of-kin. New copies of the interview guide were used for every interview, so field notes could be written down. An overview of topics discussed in the interviews is shown in Table 1. To aid understanding of complex topics, we offered participants handouts with additional information (Additional file 1). In addition, we used three scenario-sketches of fictional SCA patients. The fictional patients were given Dutch gender-neutral names to limit gender bias, inspired by hypothetical patient “Pat” from a study by Breitkopf et al. [27]. In these scenario-sketches we described cases in which informed consent and the disclosure of genetic findings can be especially troublesome for researchers. One-time interviews were conducted together by two researchers, namely MARB (PhD researcher in ethics who published several qualitative articles) and RV (MSc student in medicine without prior experience in qualitative research). Interviews with SCA survivors and next-of-kin lasted between 45 and 75 minutes and were audio-recorded and transcribed.

**Table 1:** Summary of interview topic guide (version: patient)

Interview topics
**Introduction** Introduction of the interviewers and stating of their credentials Personal conversation to build rapport. Asking permission to record and stressing voluntariness. General health since the SCA.
**Awareness and decision-making around participation** Knowledge of ARREST. *Handout 1:* Information about the ARREST study. Attitude towards medical research and reasons to participate. Potential risks of participating, including privacy concerns. *Handout 2:* List of the most important data collected during and after the resuscitation.
**Informed consent preferences** Desired level of control (opt-in, opt-out, waived). Specificity of consent and types of data. Expiration of consent, including after death. Informed consent in deceased and incapacitated patients. Views on current ARREST procedure and timing (asked for internal evaluation).
**Perspectives on return of individual genetic findings** Preferences on the disclosure of genetic findings to participants and next-of-kin. *Handout 3:* Description of four variables (treatability/severity/clinical utility/validity). The disclosure of genetic findings from deceased patients to next-of-kin.
**Special cases: three scenario-sketches** *René(e)*: who survives the SCA but becomes severely limited in understanding and communication due to neurological damage and thus unable to consent for data research. *Sam:* who passes away suddenly due to SCA and was therefore never in a position to consent for research that uses their data. *Maxim(e):* who survives the SCA and gives consent for data to be used in research. Maxim passes away some years later in a car accident: does the consent expire?
**Other aspects of data governance** Perspectives on data protection and oversight. Importance of patient engagement and participation.
**Closing** Questionnaire with socio-demographic information. Time for additional questions and remarks. Evaluation and feedback: how was the experience of being interviewed? Whether participant wants to receive transcript and findings. Recruitment of next-of-kin, thanks and closing.

### Data analysis

**Fig. 1:**
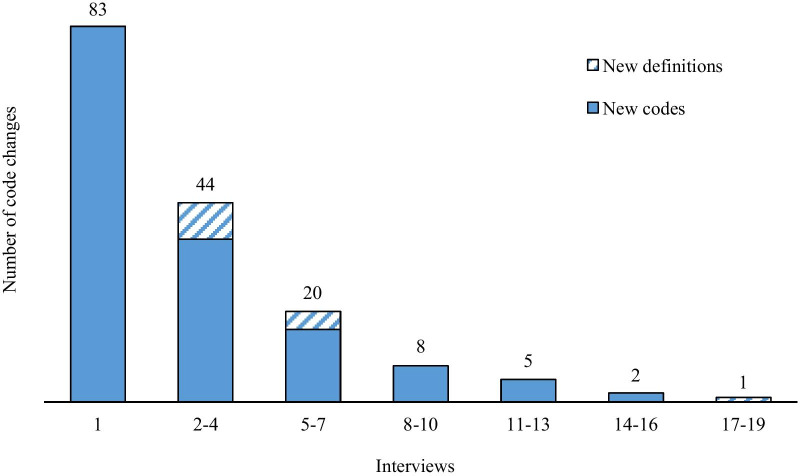
Data saturation visualized.

We analysed the interviews using MAXQDA 2018 software. The first analysis was performed through open coding by two researchers separately (RV and MARB), after which the two analyses were compared. The following analyses were carried out by one researcher and reviewed by the other. Changes to the codebook were logged after every analysis and data saturation had occurred after 19 interviews (Fig. 1). All participant names were replaced with pseudonyms. Consolidated criteria for Reporting Qualitative research (COREQ) were followed to ensure transparency and rigour of study reporting [28].

## Results

In this section we first describe the characteristics of the sampled interview population, after which we discuss three themes that arose from interviews with patients and next-of-kin: (1) risk perception, data protection and trust; (2) informed consent in acute life-threatening situations; (3) disclosure of individual genetic findings, including after death.

### Sampled population

We approached a total of 17 ARREST participants of whom 12 agreed to participate. Patients who declined (29%) did so because they were not interested, were occupied with informal care duties to relatives, or had a language barrier. Of the twelve patients, nine were interviewed at their own home and three preferred to have the conversation in the hospital. We also interviewed seven relatives or partners, some at the ARREST participant’s home together with the SCA survivor and others by telephone at a later point in time. Two of these next-of-kin were related to the same patient. None of the interviewees had any established relationship with the researchers before the start of the study. Mean time passed since resuscitation was 4.2 years (range: 0.9 – 8.8 years). Other sample characteristics are presented in Table 2.

**Table 2:** Sample characteristics.

	Patients (n = 12)	Next-of-kin (n =7)	Total (n = 19)
**Gender**			
Male	9	1	10
Female	3	6	9
**Age (years)**			
<40	2	2	4
40-59	3	0	3
60≥	7	5	12
**Educational level**			
Finished primary or secondary school	7	2	9
Finished higher education	5	5	10
**Employment status**			
Employed	5	3	8
Unemployed^a^	3	1	4
Retired	4	3	7
**Marital status**			
Married	5	4	9
Co-habiting	2	2	4
Single^b^	5	1	6
**Parents' country of birth**			
The Netherlands	11	6	17
Other^c^	1	1	2
**Relation to the patient**			
Partner	-	3	-
Child	-	1	-
Parent	-	1	-
Sibling	-	2	-

a = on sick leave, disabled or on social welfare. b = unmarried, separated, or widow(er) c = Suriname, in both cases

### Risk perception, data protection and trust

Participants thought they had granted consent for the ARREST study to “help science” and improve prevention and treatment of SCA for themselves or others. Especially since participation took little effort, the study was deemed relevant, and a personal connection with the topic was recognized, interviewees found it important to participate. Some reported feeling a need to reciprocate because of the care they received. Peter, who was resuscitated a year before and became emotional when thinking about it – for instance when discussing our deceased fictional patient Sam – stated:

Everything I can contribute for other people or other heart failure [patients], I am willing to pitch in. (…) In the end, because of research in the past, my own life was saved. That is science, and that should be developed and continued.

Some patients noted that they were happy to give their data, but in return would like to receive general study findings (“*because then you know that you didn’t do it for nothing”* – William) and thought this was a way for researchers to show their gratitude. Another reason to participate was that people experienced it as empowering. Patricia, who is working again after being resuscitated a year before, viewed participating in the ARREST study as a sign for herself that she had recovered from the SCA. An alternative form of empowerment is patient participation, e.g., in the form of patient advisory boards or patient researchers, during the design of health data research [29]. Some interviewees did *not* find such engagement important, for several reasons: they doubted that patients would have the needed research skills; views from people uninterested in this additional involvement would be excluded; or because it would take too much effort on the part of researchers.

Most interviewees had no privacy concerns associated with the use of their data for medical research. When asked what privacy meant for them, two aspects were mentioned: (1) safeguarding of personal data and (2) the right to control who has access. Health and genetic data were not considered especially sensitive and most patients were indifferent regarding the fact that their DNA had been stored, while some declared that the study was more interesting because of it (*“I think it’s very important, DNA. I think they can get a lot out of that.”* – Patricia). The only collected data that most interviewees saw as private was socio-economic information, also because the relevance of these data for health research was less clear-cut. Jessica, who is a lawyer and works with personal data herself, said she was aware of the risks but found privacy less important than the benefits of participating. When presented with the list of data collected about her, Jessica reacted as follows:

Then you know quite a lot about someone, in my opinion. Especially with these data from the Statistics office [i.e., socio-economic information], that is a lot of data. (…) I can imagine these data give you a better perspective. So actually it’s good, especially if it’s anonymous. What everyone earns shouldn’t be written here together with your name.

Concerning the need to anonymise personal data before use, none of the patients found this necessary for medical data. Some interviewees noted that in their opinion – based on experience in their work or because they thought it might inhibit medical research – privacy legislation has become too demanding. Several next-of-kin also pointed out this issue. Especially Roy’s daughter Sheila, a young woman who suffers from anxiety ever since her father survived SCA, was vigilant on this issue:

You should just be able to do your work. It’s about people’s wellbeing. I think we live with too many rules for some situations, like this one. Why would you need all those rules for the research world? You do good work. I mean, you do work for us, for everyone.

Just as Sheila mentions the “research world”, most patients preferred that their data stayed in the realm of medical research. There was less trust in commercial parties: people worried about higher insurance premiums, research findings being influenced because of commercial interests, and (genetic) discrimination from employers. In this context, Peter commented:

If I go to a job interview and they hear what happened to me, then you run the risk of them thinking: well, we’ll look out for another candidate without medical history. While I could have ridden my bike here and I’m in perfect shape. So I think, well, then I’d rather make the choice myself – whether to tell an employer or future employer that maybe, one time, I had a cardiac arrest and that I take medication.

Decision-making about whether their data would be stored and used correctly, was grounded in trust. For William, an elderly gentleman who lives with his wife and is active doing sports and volunteer work, this trust was formed mainly by what he felt was a respectful manner of approaching. Patients mentioned that they *need* to trust because they have no expertise, but also that they trust the hospital conducting the research (not the hospital where patients were treated, but well-known in the Netherlands) or the medical realm. Edward, a widower of almost ninety years old who was resuscitated six years before, did not differentiate between trust in researchers and his treating physicians:

Edward: “Well, it’s just a feeling. I don’t know what they [commercial parties] will do with it. If you know it is for science, I have complete trust. That’s very different.”

Interviewer (later): “So you mentioned trust – you said you fully trust the researchers. What is it that gives you this trust that your data are stored safely?”

Edward: “Well, the way they treated me. I’m awfully glad to be alive, as it were. They did everything to keep me alive, didn’t they? Yes, I’m very grateful for that.”

### Informed consent in acute life-threatening situations

Several patients confused the ARREST study with other investigations they took part in previously. Ruth, who experienced SCA five years before the interview, said: *“Yeah, that’s really a lot. (…) But it’s all true though, right? What’s written here? That has all happened, I think”*. Some stated they had probably forgotten because they did not mind the data being collected. Of the next-of-kin, when asked if they were aware that DNA of their relative or partner was saved and analysed, most declared that they were notified at some point, but only could recall it now because they were reminded.

Still, some interviewees preferred an *opt-in* procedure for consent from SCA survivors. People felt this would provide them the necessary control over their data, or that it was needed out of common courtesy. Patients who preferred a *waiver* of consent (i.e., no consent is required on the basis of a decision by a research ethics committee) did so because they expected these data to be solely used for research. Those preferring *opt-out* found it most important that medical research could continue unhampered. An option would be to implement *tiered* consent where patients can choose which types of data not to share: this was preferred by some but only for socio-economic data; no-one advocated tiered consent for DNA analysis.

In emergency medical research, consent from surviving patients is necessarily deferred, which was found acceptable. Regarding the appropriate timing, different views were expressed. Some shared that they were approachable as early as a few days after the resuscitation while others needed weeks or months to recover cognitively and emotionally. Patricia said:

The first few weeks I was very emotional and I cried a lot. (…) At that moment I would have also consented maybe, but I was so busy doing other things. But because [the informed consent request] came a month or two later, I was capable of thinking about it properly and I could give a sensible answer.

As for means of contact, the majority preferred consenting through a letter. Initial contact by telephone was thought to be overwhelming and unreliable as the identity of the caller is unknown, and e-mail was found impersonal and not used by everyone. The procedure as implemented in the ARREST research group was found acceptable by all respondents. Simon commented that he would be able to give a meaningful answer again after three months and that he thought *“it’s a good option to send a letter and announce that ARREST will call you. Researchers could also say: if you don’t respond it ends here. But then they can never fill their study, there has to be some pressure on it.”*

Generally, patients agreed that if opt-in consent is used, this could be a one-time, broad consent (although one participant preferred a “one-time reminder” to know that their data is still being used). More specific or tiered consent was thought to result in some patients thinking *“that is actually very confidential, maybe not that one”* (Jessica) which might hamper research participation. Others took into account the administrative burden for researchers *(“I think such broad consent would save a lot of time and paper”* – Simon) and the limiting effect on research of specific consent (*“You cannot always know beforehand for what research it is useful”* – Peter ). Moreover, a number of interviewees would find it too burdensome to be contacted with consent requests for each separate study, like the retired William who would not participate anymore if that was the case.

We also discussed in detail the situation where a research participant dies. Patients all believed that their given consent does not expire after death; some said that they assumed the data would continue to be used post-mortem even if this was not specified during the consent process. Next-of-kin were more hesitant, like Peter’s sister Claudia who felt uncomfortable by the idea that genetic data is still being used after someone passes away. Liam’s partner Sophie first thought it might be good to delete the data once it served its purpose, but later changed her mind:

No, actually I think it should just not be in the hands of the patient, because they don’t benefit from it anymore and science does, so you should look at the greater purpose.

However, many people who experience SCA die within minutes and can therefore never give consent to the use of data collected during the medical emergency. We asked whether consent (from next-of-kin) should be required in this situation; this is currently not done in the ARREST study and not required under EU and Dutch law. In contrast with participants’ views on the use of data from living but mentally incapacitated persons, where they found either opt-in or opt-out mechanisms to be required, when patients have died the majority of interviewees said notification of next-of-kin is not required; since no possibility for harm was seen whereas the data could still be useful. Simon who had an SCA around ten years ago argued that “*even after your death you can still feel useful”* and that in the future his data may be useful to validate new genetic tests. In addition, contact with next-of-kin might be burdensome and stressful for them. William’s spouse Anne said that her “mind would be occupied by other things” and that it would be alright to just use the data. Other respondents felt there needs to be some form of control by relatives, especially for genetic or socio-economic data that may implicate them as well. Peter elaborated on this:

Why do you ask consent to living persons for DNA and why do you throw it out in the open for someone that has passed way? There should be no difference between that. (…) Because it’s actually cheating, because that man or woman is not alive anymore. So you’re dealing with that disrespectfully. Certainly for the one that has passed away, but also for the family.

When we discussed whether next-of-kin are in a good position to make decisions for the deceased about continued data use, opinions were divided. Patricia doubted whether her relatives would make the same decision in her stead:

Maybe the next-of-kin don’t want the data to be used. Look, I discussed this with my husband. Not exactly this, but: ‘I will give consent, what do you think?’. He said he’d have done so too. It is my decision in the end, but if you don’t survive, that’s pretty intense for the relatives. I don’t know what they would say then

Ronald, on the other hand, thought this should be discussed with relatives and that his brother who had also experienced a cardiovascular event would be the right person to make the decision: *“My oldest brother would surely consent, without a doubt. Especially now that he’s had a similar experience himself. In that respect, it would be all right.”* His brother Martin said he would have done so indeed, but noted that consent from next-of-kin of incapacitated or deceased patients should only be sought after a few months when the family has had time to cope with the situation, because otherwise *“they could give an answer that goes against their interest, an emotional instead of a rational answer”*. Others, like the elderly Nancy whose husband had been resuscitated, felt that next-of-kin should be asked as quickly as possible:

Yes, because if you want to do that study, then the data should be collected anyway. (…) Then you get the situation that they already took it. They already got it. And I hear just now that they did so. I wouldn’t think that’s nice, so to say

### Disclosure of individual genetic findings, including after death

In the hospital where the ARREST study is based, since 2016 it is local regulation that to participate in genetic research, people need to agree that they want to receive results on clinically actionable genetic findings. Before then, a participant could opt to donate DNA but not be informed on any individual findings. Almost all ARREST patients sampled for this interview study were recruited after 2016 and had therefore agreed to the new policy. However, one of these patients, Ronald, when asked did not want to be informed anymore of any individual findings:

No, because I’m feeling well. I probably just came back from the hospital at that time. Then you don’t feel a hundred percent yet and then you probably think differently about it. But at this moment, I say: no.

Another patient also changed his mind. After being resuscitated in 2010, Simon had declared that he did not want to be informed on genetic findings, but said during the interview that at present he would like to be informed if anything clinically actionable was found. He did not recall making this choice:

Interviewer: “Can you imagine why you answered ‘no’ to that question in the consent form?”

Simon: “No, it is such a long time ago. The first year after I came home from the hospital, I had some strange things. Coffee mugs that I had to clean, I put in the fridge instead, and open milk cartons I left in the pantry. My wife thought: this guy is driving me crazy. (…) In the first year, things like that happened a lot. Your brain really needs some time to recover. So I really wouldn’t know.”

Neither of them had ever contacted the ARREST researchers to report their change of heart. All other patients wanted to be informed of clinically actionable genetic findings as per their consent – of note is that a number of these participants had forgotten that their DNA was analysed. Patients recognised a potential conflict of interest: a patient’s ‘right not to know’ genetic findings but also a duty for researchers to report findings to those who *do* want to know. Whether the finding was incidental or deliberately sought after by researchers, i.e., related to the field of SCA or not, was not seen as important. We also queried if the interviewees would prefer that DNA is extracted from left-over blood routinely drawn in the hospital for critical care or rather have additional blood sampled after consent. Our participants would prefer the use of residual blood (current practice in ARREST), even though the blood is stored without the patient’s knowledge until consent procedures are started.

To discuss reasons for wanting to receive individual genetic findings, we presented participants with four variables: risk, severity, clinical utility, and clinical validity. Some interviewees noted that risk and clinical validity would be most important since these factors determine how to evaluate the other two, but generally the main factors were clinical utility and severity of the condition, which were often mentioned together. Roy, who is retired and lives with his wife and daughter, said that he would like to be informed so that he knew what symptoms to look out for. Other reasons mentioned were to be able to warn their children to check for risks, or because knowledge about genetic determinants could give some peace of mind, as Liam explains:

Yes, definitely. The reason that this [cardiac arrest] doesn't bother me anymore in daily life is that the blood clot was taken out and they explained to me what had happened. What had gone wrong in my body. I could see it clearly on the monitor during the catheterisation. So you finally know what it was that made you feel unwell. That was really nice. So in ninety percent of the cases I’d say: tell me everything you can find about me, please.

A difficult situation in SCA research is the case of deceased patients whose DNA is analysed but who are not alive anymore to share potential genetic findings with their relatives. Using the fictional case of patient Sam, we asked whether relatives should be informed of DNA analysis. This is not required under Dutch law but sometimes done by researchers through the family’s general practitioner. Patients and next-of-kin believed that when genetic data continues to be analysed after death, it is right to inform the relatives and give them the choice whether to be informed about potentially clinically actionable findings. Next-of-kin stressed that the manner of approaching should be sensitive and tactful, and Liam’s partner Sophie emphasized that researchers *“should make it clear how this data is being handled and why it’s important that the research is being done”*.

Lastly, with the case of Maxim, we explored if participants felt that family should be informed of potentially relevant genetic findings after the participant had died, even if she/he had stated the desire for this not to happen. Opinions were divided: while some thought privacy and the patient’s wishes should be respected after their passing, others looked at it in terms of beneficence and found the value for relatives’ health more important. As a sister of someone who suffered from SCA, Claudia stated:

If I look deep into my heart, I think it expires. [Being able to prevent] is more important than to respect the wishes of a deceased person. But well, it’s also dependent on what is found. It depends on how life-threatening it is.

Middle grounds were also suggested: providing the information if asked for by relatives, but stressing that it is against the deceased’s wish; or reporting only findings that suggest a very serious threat to relatives’ health.

## Discussion

This qualitative study explored the views of Dutch patients and their next-of-kin regarding health data research in the acute care setting. Hereafter we discuss in turn three observations of ethical concern for research with personal data of these particularly vulnerable subjects, namely: first, the distinction created between the medical realm compared to other areas, and trust as basis for health data privacy; second, the lack of awareness among patients about the contents of their given consent; and third, the ethicality of the use of data after death. The explicit aim of this study was to obtain participants’ moral intuitions and gather information about experiences donating data to a particular SCA study in the Netherlands, in order to inform policy but without drawing definitive normative conclusions on specific moral dilemmas: elsewhere we will “cross the bridge from is to ought” [30].

### Privacy and trust in the medical realm

Health-related research data was not seen as especially sensitive and donating was viewed as part of being a good citizen who contributes to medical science. We can link this to an idea described by sociologist Miriam Ticktin [31] who argues that the current medical paradigm (where health-care and -research are always in the name of the good) connects people by virtue of the fact that we are all potential patients, i.e. ‘the universal sick body’. Similarly, some interviewees believed that data protection rules should be less strict for medical researchers who “work for *us*, for everyone”. Participants became worried only when data moves outside of this medical sphere, which is in line with scholarship on privacy that emphasises contextual integrity [32, 33]. People want to give their *health data* for *health-related uses*. Because people struggles to see the connection between medicine and environmental factors., data relating to one’s socio-economic status was regarded as sensitive and deserving of other contextual norms, i.e., stricter consent and data protection requirements. In contrast, while the Dutch ARREST study provides a separate (‘tiered’) consent option for DNA analysis, no interviewee advocated this. Likely because participants regarded genetics as part of the “medical research world” they did not support such genetic exceptionalism, i.e. the idea that genetic information deserves special protection compared to other types of medical data [34]. Our study thus suggests that knowing the utility of the data is important for patients’ views on the acceptability of research and that consent procedures may need to include explanations when collected data is not obviously connected to health care. However, one could argue that especially with big data analysis, all personal data is in essence health-related [35, 36]. Further ethico-legal study is needed on the distinctions between normal and special categories of data.

In addition, we found that patients’ participation in the observational ARREST study was grounded in *trust*, which corresponds with results from research investigating SCA survivors’ views about clinical trials without prior consent [37] as well as with findings from a number of publications about health data research [38–41]. Lea & Nicholls [42] have argued that the traditional physician-patient relation forms the basis of trust in the medical realm, and that health information governance should honour subjects’ expectations about this relationship to avoid undermining it (e.g., causing patients to withhold information important for their treatment because of wrong uses of health data elsewhere). Similarly, in our study sample, some interviewees’ (misplaced) trust in academic researchers came from having been treated well earlier by physicians not involved in the research. Trust has been defined as a “willingness to be vulnerable” [43] and this willingness may be higher among those who survived acute and life-threatening illness. Trust seemed to stem from a universally recognised trustworthiness of publicly funded medical doctors and researchers as opposed to commercial parties operating for profit [44–47]. However, health data research is becoming increasingly interdisciplinary and based on public-private partnerships, so that the distinction between medical and non-medical uses becomes ever more difficult to make [48]. Research is required on these divisions between realms and on the conditions for commercial uses of health research data, as well as on the concept of vulnerability in relation to health data research.

Trusting might not always be a voluntary act: a *need* to trust may derive from a lack of knowledge. Biobanking participants generally have limited understanding of the aspects of research included in consent forms, and the level of understanding depends on contextual and demographic factors such as education [49]. A recent deliberative study showed that better informed persons are more supportive of sharing data for health research [50]. As such, improving research participants’ knowledge on data protection processes (e.g., through public engagement, increased transparency or more extensive consent procedures) might enhance autonomy but also improve patient trust. Especially in emergency medicine where prior notification of data collection is impossible, guidance is needed to promote the contribution of lay people’s expertise to patient and public engagement (PPE) initiatives, since their actual influence is limited [51, 52]. However, doing so also increases costs for researchers, potentially brings about disparities among participants with differing capacity for understanding, and “might transfer an unwelcome sense of responsibility to patients” [53].

### Consent or non-conception?

Even though health data were not regarded as particularly sensitive within the medical realm, some interviewees preferred an opt-in procedure, i.e., the reverse of the well-known privacy-paradox where people care about their online privacy but do not take action to safeguard it [54]. Requiring informed consent may lead to lower participation and a biased sample [55, 56] and some authors have argued that people have a social duty to participate in low-risk research [57, 58]. However, trust is something that is created and in our setting, this may currently require explicit informed consent: as a gesture of courtesy and to enable some control over the usage of one’s data. Findings from literature in non-acute settings vary. In a study on waived consent for research using data from general practitioners (GPs) in the United Kingdom, participants did not accept the waiving of consent, for the same reasons we found [59]. In a more recent study, roughly three out of every four interviewed German outpatients stated they approved research with clinical data without consent [60]. Since views differ between different times and localities and depend on the types of data collected and the specific healthcare setting, policies would need to be sensitive to context, although this could complicate international harmonisation of data [61, 62].

In acute care research, consent is necessarily deferred. This deferring of consent from SCA survivors was seen as acceptable. The appropriate length of deferment was largely determined by the length of situational incapacity, since recovery for SCA survivors is hindered by a range of physical, emotional, cognitive and social challenges [63]. Within the ARREST study, a time period of three months was thought to be appropriate, but we recommend further normative study on this issue and suggest leaving room for an individualized approach due to the differences in recovery time. Of note is that we refer to ‘deferred consent’ which for interventional research might be more aptly called ‘research without prior consent’ (RWPC) as study procedures already took place. There is an on-going debate on whether the deferring of consent is appropriate in clinical trials [64–67]. However, this is not necessarily the case for observational studies where data and samples might already be collected but are retained, for instance, by a Trusted Third Party (TTP) before release to researchers.

In our study all patients approved of a ‘broad’ type of consent, a practice which is debated among bioethicists but increasingly necessary when big data is used, and appears permissible under the GDPR [68–70]. Those who want to have the autonomy to choose whether to donate data are willing to leave the implementation into research to the investigators. This might explain the lack of awareness we encountered among participants about the contents of their given consent, which corresponds with findings from a study about cancer genetic research where most patients remembered participating in interventional research but 67% did not remember contributing to database studies [71]. The fact that participants had forgotten their indicated preferences regarding the disclosure of individual genetic findings, raises questions about the validity of their consent for genetic analysis. In interventional research, the existence of a ‘therapeutic misconception’ (i.e., participants believing that the research will clinically benefit them) may limit patients’ ability to give valid consent [72]. In biobanking and genomic data studies, however, the boundary between research and care is known to become increasingly blurred due to the disclosure of individual genetic findings, and this risk of therapeutic misconception is therefore less relevant [73]. In contrast, our study provides evidence of a therapeutic *non-conception* among biobank participants: a term coined by Tupasela & Liede [74] meaning that “people are not aware of how their samples and information are being used” (p. 269) while lack of awareness and up-to-date preferences regarding DNA analysis may have implications for participants’ health. Namely, this may limit the possibility for acting on individual findings or, conversely, receiving these findings may give rise to anxiety or stress.

It is up to debate whether participants should be asked again every few years if they want to receive genetic findings. In recent years, a ‘dynamic consent’ process has been debated where participants can update their preferences periodically or when the data are used for new aims outside of the original consent [75–77]. Our interviewees believed that consent is not subject to a certain shelf-life, consistent with other studies investigating patient views [78]. However, the impact of receiving genetic findings on people’s wellbeing may suggest otherwise for studies that collect DNA and re-contact participants (on an *ad hoc* basis) with clinically relevant findings. While potentially reducing the number of study participants over time and creating additional costs for researchers, a periodic renewal of consent would offer participants a chance to continuously exercise their rights [79]. Further study is needed on the ethical appropriateness of such a policy, and on what findings should be disclosed to patients with acute and critical illness like SCA, since judgments about the clinical significance of genetic results differ between medical specialties [80]. Of note is that a dynamic consent policy could only be implemented when both *the right to know* and *the right ‘not to know’* about genetic results are respected [81]. In some cases, a right not to know puts researchers in a morally uncomfortable position, as was described in a study on a genetic subtype of a certain cardiomyopathy where median age to death in men was 41 years but researchers were not allowed to approach individuals at high risk who had refused to receive their results [82]. A full discussion of the rights related to disclosure of genetic research results is outside the scope of this article.

### Data protection after death

If consent is currently not confined to an expiry date, does it also stay in effect after the participant’s passing? Although the extension of consent beyond death is often unspecified in the consent procedures for biobanks and registries, as it was in the ARREST study, most interviewees thought it obvious that data continues to be saved and used after death, because the person granted consent while he or she was alive and fully aware. However, the majority of SCA victims do not survive the event and have never given consent for the use of their data or genetic samples obtained during emergency care before the onset of death. Especially with the rise of biobanking and longitudinal studies, the contribution of deceased people to the pool of (big) data for research is increasing, and non-use of data from these deceased patients may lead to biased studies [83, 84].

The GDPR and the Dutch implementation act do not apply to deceased persons, but national civil law does include provisions to allow the use of data for research without consent when a patient has died [85]. Thus, in the ARREST study no consent is asked from next-of-kin. While some respondents disagreed with this policy, others said data should be used without consent, for reasons similar to those mentioned by interview participants in other disease areas [86]. Interviewed next-of-kin seemed more uncomfortable with the idea of post-mortem data use than patients themselves. In addition, some patients were not certain whether their next-of-kin would have made the same decision as they had. Whether there is truly an inconsistency can only be established with additional quantitative research.

Concerning genetic data, all interviewees thought that next-of-kin should be informed about the fact that post-mortem genetic data analysis is taking place and be given the choice to be contacted about findings with potential relevance for their own health, if no prior preferences had been reported by the deceased. This is similar to a large quantitative study where over 80% of biobank participants thought such post-mortem disclosure was acceptable [27]. European Commission recommendations on genetic testing recognise a right of access to samples and data from a deceased person when blood relatives’ health is at stake [87]. Chan et al. [88] have argued that studies that disclose individual genetic research results to participants should also do this for relatives of deceased participants, at least when asked, but potentially also actively. Still, return of these findings to living relatives of research subjects is not standard practice in many places and further discussion is needed about whether it should be, and if so, about how this could be done in a sensitive and trust-promoting manner. Also, opinions were divided about whether a living patient’s wish not to inform family members of potentially relevant clinical genetic findings (a wish whose ethicality is already subject of debate due to the familial nature of genetic information [89]) should be respected after death. This inconclusiveness is consistent with literature on this topic [90]. In the end, many of these issues come down to the right balance between respecting the deceased person’s wishes on the one hand, and the rights of living relatives or partners on the other: a question that deserves renewed empirical and philosophical study given the increased technological possibilities (e.g., around data storage or DNA analysis) and the heightened connectedness of personal data with ourselves and others [91].

## Study limitations

Health data research is a complex topic. Despite the use of handouts and scenario sketches, some themes may have been difficult to understand for participants, which potentially biased our analysis towards those interviewees who were able to formulate a coherent opinion. We also interviewed individuals relatively long after their arrests occurred: while this provided valuable insight on long-term study recollection among participants, we wish to stress that these findings may not be generalizable to people who consented to participation more recently. Discussions about the trustworthiness of researchers and data safeguards might have been influenced by the perceived reliability of the researchers in our interview study, especially because these researchers are affiliated with the cardiac arrest registry to which patients had donated their data. While we aimed to include a diverse group of patients, people of ethnic minority were underrepresented. What is also missing from our paper are the perspectives of next-of-kin of deceased patients, and of patients who had declined to give consent to data donation for research. After talks with our institution’s REC we decided not to include these groups, not only because they are difficult to identify but also because approaching them is challenging in terms of ethical and legal constraints. Future studies should aim to include those groups as well as promote a diverse mix of participants, especially in terms of ethnicity.

## Conclusions

In this qualitative interview study we have explored the views of sudden cardiac arrest survivors and their next-of-kin concerning the processing of health and genetic data for research purposes. Our findings suggest a blurred boundary between research and care, given that data donation for medical research was based on trust in the medical field in its entirety. We also highlight questions about the acceptability of a one-time consent and about responsibilities of patients, researchers and ethics committees regarding data governance. Our study provides a first step toward the creation of an empirically informed ethical framework for (big) data research in acute care settings. Other stakeholders’ perspectives as well as substantive ethical analysis should also be included in order to build on more than the patient perspective alone. Further study is needed regarding the observed distinctions between different types of research data, the desirability of further information provision to enable participant empowerment, and the questions around the research use of data after death including the return of genetic findings to relatives.

## Supplementary information

**Additional file 1.** Interview guide and handouts.

## Data Availability

Not applicable.

## Abbreviations

ARREST: AmsteRdam REsuscitation STudies
COREQ: COnsolidated criteria for REporting Qualitative research
CPC: Cerebral performance category
ECG: Electrocardiogram
ESCAPE-NET: European Sudden Cardiac Arrest network towards Prevention, Education, New Effective Treatment
GDPR: General Data Protection Regulation
GP: General Practitioner
PPE: Patient and public engagement
REC: Research Ethics Committee
RWPC: Research without prior consent
SCA: Sudden cardiac arrest
TTP: Trusted Third Party

## Footnotes

1 We use the word ‘patient’ instead of the more neutral ‘person/participant’ in order to differentiate between SCA survivors and next-of-kin.
