# Supplementary material for: Health data research on sudden cardiac arrest: perspectives of survivors and their next-of-kin
Source: BMC Med Ethics. 2021 Jan 28;22:7. doi: 10.1186/s12910-021-00576-9 (PMC7844916; doi:10.1186/s12910-021-00576-9)
Supplement: Supplementary file 1 — Additional file 1. Interview guide and handouts. [file 12910_2021_576_MOESM1_ESM.pdf]

Amsterdam UMC
Universitair Medische Centra

Interview guide for patients (translated from Dutch)

Introduction

- ➢ Thank you for coming, my name is [...] and work at the AMC Department of Cardiology and the department of Medical Ethics.
  If the interviewee asks what ethics is: Ethics is the study of 'right' actions, i.e. what is right and wrong. In medical ethics we examine what doctors or biomedical researchers should do in certain difficult dilemmas.
- ➢ Before I start with the questions, can you tell us a little about yourself?
- ➢ I have here a list of questions regarding the use of personal data for medical research. The purpose of our research is to map your opinion on these topics so that we can improve medical research. It's really about your opinion, there are no right or wrong answers. The interview will take about an hour to an hour and a half.
- ➢ Everything said here in this room is confidential and will only be shared with other researchers involved in this study. The results will be published completely anonymously (without name, without address, without date of birth, etc.).
- ➢ The research is entirely voluntary. You can still decide to stop at any time.
- ➢ Do you have any questions? You can also ask questions throughout the conversation.
- ➢ For the investigation it is necessary that we record this conversation, so that we can listen to your exact answers at a later time. This is of course also anonymous. Are you okay with that?
- ➢ The recording of the interview is shared encrypted with a company that transcribes it into a transcript and has signed a nondisclosure agreement. Are you okay with that?
- ➢ [turn on recorder]

Motivation and ARREST study

- ➢ How are you now?

Sometime after your resuscitation, you have given permission for your data to be used for the ARREST study.

- ➢ What do you remember of this?
- ➢ Do you have some idea what the ARREST study does?

The investigator hands over the handout card [1, provided in this document after the interview guide] with a brief explanation of the ARREST study and the current interview study, which the interviewee can read through.

- ➢ What are reasons for you to give consent for research?
  - o And for the ARREST study specifically?
  - o Do you see any risks in giving consent to the use of your data for research?
  - o Are there any conditions which an investigation should fulfil do before you choose to give consent?

Privacy

Privacy has been increasingly in the news in recent years. Last year, a new law was also introduced in Europe that protects the privacy of personal data, the General Data Protection Regulation (GDPR).

- What is your definition of privacy?
- Is privacy important to you? Why (not)?
- Did you consider your privacy when you agreed to participate in the ARREST investigation? If so, do you remember what your considerations were?

The researcher provides a handout [2] with a list of all the different data that were collected for the ARREST study *before* the person was asked for consent.

- How do you feel about this when you see the data sources listed like this?
- Did you know that this information is stored ?

The researcher explains that it is possible that less data has been stored with the interviewee than is stated here on the card. For example, the socio-economic factors are new in the ARREST study. [Researcher explains what these are and why they are collected]

- How do you feel about socio-economic data being collected?

In the ARREST study , data can be shared between researchers if necessary.

- Do you think it is important that personal data such as name, address and date of birth are deleted before researchers share this information with each other?

Medical information, such as this one on the list, is also often shared internationally in order to conduct research on larger groups of people.

- What do you think about this? What do you think of research in which data is shared internationally compared to research that is only carried out in the Netherlands?

Medical information can also be shared with commercial parties such as the pharmaceutical industry or insurance companies. The researcher states that ARREST researchers would never do this without the person's consent.

- What do you think about this?
  - Do you have a different view on the sharing of personal data with government agencies or universities compared to commercial parties such as the pharmaceutical industry or insurance companies?
  - And what if your employer had access to these data?
  - Who should really *not* gain insight into your data from the ARREST study?

Indicate that ARREST researchers secure the data as well as possible and share with no one other than researchers without the explicit consent of the participant.

- What gives you confidence/trust that your ARREST study data is securely stored?

Informed consent

The next theme is about asking people for permission to participate in medical research. The list we showed earlier has all the different data currently being collected for the ARREST study before permission could be asked. This is all data that was already there (for instance, information about your heart rhythm collected from ambulance personnel), but that may have been deleted if the researchers had not asked for it. It is possible that slightly different data was secured with you at the time, this may differ over the years.
Asking consent usually goes through an opt-in or opt-out procedure. In an opt-in procedure, a person must have given explicit consent before they can contribute to research. In an opt-out procedure, a person must actively object to the investigation if he or she does not want to participate. If the person does not object, they will automatically participate.

- Which procedure had been most appropriate for the ARREST study and why?:
  1) The researchers did not have to request permission for this study at all
  2) Opt-out, i.e. without objection, the person automatically participates
  3) Opt-in, i.e. the person must first actively consent

It has been decided that during the ARREST investigation permission must be requested for the storage and use of this data. When you had your cardiac arrest, a few months later you were asked permission for the use of your data for scientific research in cardiac arrest, but it was not specifically explained what studies run exactly and what they are investigating. We call this broad consent.

- What do you think about that?
  - Which would you prefer: to be informed in advance of the precise studies that are underway and be able to give permission for specific studies to use your data? Or broad permission such as with ARREST, where you give consent for research in a certain field, but do not see the specific studies?

Since research data is now stored electronically, it can theoretically be kept forever. Sometimes new research uses for data are invented long after consent has been given by the individual to use the data.

- *If your already stored data qualify for a new study, for example in 10 years, do you think researchers should always ask for permission again?*
  - Is there an end date attached to the consent?

- If another field that has nothing to do with cardiac arrest, such as cancer research, also wants to use this data for research, do they have to ask for permission?

- Looking at the list from earlier, would you like to be able to individually choose per section which data you want to share and which not? Why (not)?

The tricky thing about the ARREST study is that it concerns people in an emergency situation. The researchers cannot request permission from the person at the time of emergency. Therefore, requesting permission is delayed until after resuscitation.

- *If not addressed:* Do you remember how you were approached at the time for the ARREST investigation after your resuscitation?

At the time, you were contacted by letter and telephone to ask whether this personal data may be used for research.

- How did you feel about the way you were approached to participate in the ARREST investigation?
  - Do you remember if you read the letter in its entirety?
  - Do you remember if the information in the letter was clear?

- What was the best timing for the investigators after your resuscitation to approach you for permission to use your data for research? Why?

- What kind of approach to requesting permission for the study would you have liked best? For example, by telephone, letter, e-mail or if a researcher came to see you in person while you were in hospital? Why?

The exact consent procedure of ARREST today looks like this. It may be that the procedure was slightly different when you were asked for permission. Persons eligible for the ARREST study now receive a letter at home, three months after resuscitation, from the Ambulance Service that transported the patient to the hospital. It states that the Amsterdam UMC will contact you by telephone to participate in the ARREST study. The person then has 4 weeks to object to this by means of an enclosed form that can be returned , i.e. opt-out. If the Amsterdam UMC does not receive an objection, the person will be called. After the person has given permission by telephone to participate , another letter will follow with a written consent form , or opt-in. If it is subsequently signed and received at the Amsterdam UMC, the person has given permission.

- What do you think of this procedure?

Genetics

When doing medical research, many different types of data are saved , as seen on the list. At ARREST also genetic information is examined, this is also known as DNA . DNA is the collection of all hereditary material that a human has. When you have a child, you give them half of your DNA. The DNA is examined to look for new, unknown abnormalities that may be related to the development of sudden cardiac arrest. This is done to gain more insight into how cardiac arrest occurs, because it has a very complex pattern of causes. DNA appears to be just one of the factors that can contribute to cardiac arrest. An unhealthy lifestyle, such as smoking, also contributes to this.

- When deciding whether or not to participate in a medical examination, is it important to you whether DNA is also stored?
  - Why (not)?

The DNA is extracted from 'residual blood' in ARREST. (If not discussed: During hospital admission after resuscitation, blood is always taken, which is necessary for certain tests before the treatment. Sometimes a few milliliters of blood remain and DNA can be determined from this residual blood.) ARREST stores this residual blood until (several months later) permission for DNA analysis is asked. Thereafter, the actual DNA takes place. The disadvantage of this is that the blood is stored for a while without the person knowing. Another option would be not to keep this blood, but to ask people who have had a cardiac arrest and give permission for DNA testing to come back and give blood.

- What would you rather have: DNA test from 'residual blood' or come back again to have blood taken for DNA testing?

<u>Reporting genetic findings</u>

Some diseases are caused by abnormalities in the DNA. These can be very different diseases, for example harmless but also very serious. It may be that while studying the DNA for medical research, DNA abnormalities are found that indicate that the risk of a certain disease is increased for the person .

- Would you like to know if researchers find a DNA abnormality in you that increases the risk of a disease ?
  - Or does that depend on something?

The researcher shows the person 4 cards with 4 factors written on them [handout 3]: the treatability of the disease, the severity of the disease, the risk of the DNA abnormality or the reliability of the test. These are explained orally .

- Can you indicate which of these 4 factors you consider most important in deciding whether or not you want to know the results of your DNA test?
  - And which factor is most important to you after that and why?
  - Why are the other factors less important to you?

The DNA abnormality found may be related to the study: For example, an increased risk of cardiac arrest in the ARREST study. However, it may also be that researchers find something else, entirely coincidentally, that has nothing at all to do with this field, for example an increased risk of breast cancer.

- Do you think differently about abnormalities found by chance, such as an increased risk of breast cancer compared to abnormalities that the researchers were really looking for, such as an increased risk of cardiac arrest?

- Do you think that investigators have a duty to report DNA deviations to the person concerned?

Some people indicate that they would prefer not to know if they have a DNA abnormality. Other people really want this.

- Do you know what happens in the ARREST study when people do not want to know the results of their DNA tests?

For a number of years now, the AMC has had the policy, including for the ARREST study, that a person who wishes to participate in research with genetic material must also agree to receive the results of any genetic findings. The AMC has chosen this policy because they do not want to give researchers potentially important knowledge about a person's health, on which they cannot act.

- What do you think about that? Do you think that a person has the right not to be informed of the result of the DNA test, if he so wishes? Why (not)?

Sometimes a DNA abnormality can also affect family members of the affected person, as they partly share the same genes and therefore possibly have the same DNA abnormality.

- Do you think researchers should notify family members about DNA abnormalities?

Some people would not want family members to be informed that DNA testing has been done on them.

- Do you think that someone who donates DNA for scientific research should be allowed to decide for himself whether family can be informed?

<u>Deceased and vulnerable patients</u>
We go to three hypothetical (not real) scenarios in which it is difficult for researchers to ask for permission request for research on cardiac arrest and that involve dilemmas arise regarding the reporting of genetic findings. The first scenario. Imagine if a fictional person named René has had cardiac arrest and survives CPR. René has been in hospital for a while and they find out that René suffered brain damage during the cardiac arrest. René is therefore very limited in his understanding and communication. René has a partner, children and also a sister. Because René suffered brain damage, René unable to give consent to participate in research.

- What should ARREST and the investigators do in such a situation and why?
  Present the following three options:
  1) Delete the data.
  2) Request permission from legal representative, next-of-kin, or closest relative.
  3) Use the data without asking for permission.

  If the second option is chosen:
  - Which person is most appropriate for this?
  - What is the best timing to approach the individual to ask for consent?
  - Should this be done using opt-in or opt-out?
  - And what if family members have different opinions?

Another difficult part of research on people who have had a cardiac arrest is that many of the people unfortunately do not survive.

- What do you think about the use of data from deceased persons for research?

I would also like to illustrate another case, namely of Sam. Sam is resuscitated after cardiac arrest, but unfortunately does not survive. Because Sam is deceased, Sam can no longer give permission to participate in research. Sam has a partner, a brother and a sister.

- What should ARREST and investigators do with Sam's data and why ?
  Provide the same three options.

  If the second option is chosen:
  - What is the best timing to approach the individual to ask for consent?
  - Should this be done opt-in or opt-out?

In the Netherlands it is arranged so that data of deceased people may be used for medical research under certain conditions, without having to ask consent from next-of-kin.

- What do you think about this?

Imagine if Sam had not been an adult, but was a child who died of sudden cardiac arrest.

- How do you feel about the use of his or her data in this case?
  - How should researchers deal with the data in this situation? Is this different for a child than for an adult?
  - If option 2: What would be the best timing to approach parents?
  - Indicate that for ARREST, if it concerns a deceased child, parents are always asked for permission for use of the data. Fortunately, this rarely happens.

Let's go back to adult Sam. On Sam's blood researchers also performed DNA analysis as part of the study. We assume that family members have not been informed about this, as in the Netherlands no permission from a family member is required. Researchers have found a risky DNA abnormality. Sam has a brother and a sister who might also have the abnormality. Sam 's family members may want to know , but are unaware that DNA testing has been performed.

- Should family members be notified?

I will ask the next few questions based on Maxim's fictional case. Maxim has suffered cardiac arrest and is resuscitated. After that, Maxim was asked to participate in the ARREST investigation, for which Maxim has given permission. Sometime later, Maxim unfortunately dies in a car accident.

- In the ARREST study, what do you think happens to Maxim's data after death ? *Explain what happens in the ARREST study .*

- What do you think about the use of data of a deceased person who has previously given permission for the research?
- Do you think that people should be allowed to decide for themselves what happens with their data after death?

Maxim has three brothers and sisters. DNA research was also carried out for Maxim as part of the study. Researchers have found a risky DNA aberration, which Maxim's siblings could also have. Maxim has indicated that he / she does not want to know the result. Maxim has also indicated that he / she would rather not have family informed that a DNA test has been done, but Maxim has now passed away. The researchers don't know if Maxim's relatives might want to know .

- Do you think that Maxim's wish that family members are not informed about the DNA test should also be granted after Maxim's death ?

Governance and oversight

Besides individual control based on consent, there are other ways to ensure that data will be handled well. First is for example an ethics committee that checks whether new research plans are ethical. We call this a Medical Ethics Review Committee. Many hospitals, including the AMC, have such a Medical Ethics Review Committee.

- Did you know that this committee existed?
- How do you feel about the existence of such a committee?

There are basically two different types of medical research. Some studies conduct experiments on humans, for example testing new types of medication. In other studies, such as ARREST, only data is included in the study and the person does not actually undergo an experiment. The person does not have to do or undergo anything in order to participate.

- Do you think there is a difference between these two types of studies in terms of the review they should undergo by a medical ethics committee?

If not discussed: *Some people find that studies use previously measured data, such as ARREST, less stringent or not assessed need to a Medical Ethical Committee.*

- What do you think about this?

In addition to individual consent and a medical ethics review committee, another way to ensure proper data handling is more patient involvement in scientific research.
People can participate in medical research at many levels. Usually, individuals only provide data and consent to the use of this data for research. People could also become even more involved in research by, for example, asking them more regularly for advice on what kind of research we should do or what the data should be used for. This could be done in patient committees, by means of interviews or with advisory boards. At a further stage, individuals or patients could even contribute to the research itself by designing a study, conducting experiments, or analyzing data.

- Do you consider patient participation in scientific research important?
- Do you think it is generally important that patients be informed of the results of the research to which they have contributed ?
- What level of involvement do you think would be best in scientific research?
    - And do you see any disadvantages in that?
- And what would you like yourself?

Closing

- Ask to fill out questionnaire on general data (date of birth, etc.).
- Are there any things you wanted to say that we didn't get around to?
- Do you have any questions?
- What did you think of the interview?
- We will convert the recorded audio to text. Would you like to receive a copy?
- Would you like to receive the results of the survey in its entirety when all interviews have been done? Keep in mind that it can take a long time before the investigation is fully completed.
- As you have probably noticed, some of these questions also relate to your loved ones, for example when it comes to DNA or about giving permission for research in certain cases. We are therefore very curious about the opinion of loved ones of people who have had cardiac arrest on some of these topics. This shorter interview of a maximum of 45 minutes can take place by telephone. Do you have any loved ones who might be interested in getting involved? If so, could you ask them to participate and let us know? Then we will contact them to make an appointment.
- Here you have our contact details. You can reach us on this general number if you have a loved one who is interested. Then you can leave his or her telephone number and we will call them. Or if you have any questions later, or anything to share, you can reach us on this number as well.
- Thank you very much for your contribution.

Amsterdam UMC
Universitair Medische Centra

Examples

Source	Data

Before obtaining the patient's consent

**Ambulance service**	Heart film (ECG), times of departure and arrival of the ambulance, address of resuscitation, information about the course of the resuscitation (e.g. blood sugar measurement, heart rate, length of resuscitation).
**Automated External Defibrillator (AED)**	Heart film (ECG), time of defibrillator connection (or AED) and time of 1st shock
**Dispatching room ambulance service**	CPR location and time, name, surname, address, gender
**Treating hospital**	DNA (from residual blood left over from treatment), survival

After obtaining the patient's consent

**General Practitioner**	Medical history
**Pharmacy**	Medication use
**Netherlands Statistics**	Household income, country of birth of person and parents, capital (anonymised)
**Treating hospital**	Information on diagnosis and treatment

ARREST study (AmsteRdam Resuscitation STudies)

The ARREST research group studies out-of-hospital cardiac arrest in the Netherlands. By examining data from people who have had cardiac arrest and analysing how CPR works, more insight can be gained into the causes of sudden cardiac arrest, and which factors influence survival after cardiac arrest. Medical information is required to carry out this examination, including data obtained from the ambulance that transported the person and the hospital that treated the person.
After you were resuscitated, you were also asked if you would like to contribute to the ARREST study by releasing this information about your treatment for research. At the time you indicated in writing that researchers could use your data regarding your resuscitation and that you could also be approached later for other research.

Interview study

Since you indicated that you could be approached later, you have recently been called about a new study, set up by the Department of Cardiology in collaboration with the Department of Medical Ethics. This interview study, in which you participate today, examines the opinions of individuals who have had cardiac arrest. In particular, the interview will be about storing and using personal medical data from your resuscitation for scientific research.

RISK<br>of developing the condition<br><br>*A DNA defect can be one of several causes that together lead to the development of a disease. Depending on the DNA defect and the disease, this risk can be low or high.*	TREATABILITY<br>of the condition<br><br>*Diseases caused by DNA abnormalities can differ in the degree of treatability: some diseases can be treated well, others cannot be treated.*
SERIOUSNESS<br>of the condition<br><br>*Different illnesses can be different in severity, from harmless to life-threatening. This also applies to diseases that are (partly) caused by DNA abnormalities.*	RELIABILITY<br>of the test<br><br>*Some tests provide information about a disease with 100% certainty, but some tests are less certain, for example, information about the probability that a DNA defect will lead to disease.*
